# Belle Vue Hospital, New York

**Published:** 1893-07-15

**Authors:** 


### The Institutional Workshop.

### WITHIN THE HOSPITALS.

### BELLE VUE HOSPITAL, NEW YORK.

(By our Special Correspondent.)

Founded in 1811, this hospital contains 780 beds, of which 750 on an average are constantly occupied. The medical staff numbers no less than 34 physicians and surgeons. Belle Vue is, in reality, the great poor-law infirmary of New York City, the qualification for admission being one year's residence in the city or county of New York. In practice, every case of illness and accident, and all mental cases which come under the notice of the police and local authorities, are sent to Belle Vue, failing other suitable accommodation. The institution is managed by the Board of Public Charities and Correction, of which Mr. H. H. Porter is the able and courteous president. In these circumstances Belle Vue cannot fail to be a hospital of surpassing interest to foreigners who visit the United States. Here, if anywhere, a reasonable judgment may be formed of the adequacy of the provision made by the municipality for the sick poor, and of the character of the system for which the Board of Public Charities is responsible. Every class of illness, disease, and accident finds its way in due course to the wards of the Belle Vue Hospital, which necessarily presents a splendid field for clinical research and investigation. The medical staff includes the names of the most eminent physicians and surgeons of New York City, and the resident medical officers are selected by competition from the whole body of students attending the New York medical schools. The nursing is undertaken by the Society of the New York Training School for Nurses, which has sent forth an army of able graduates, who have made this school deservedly famous all the world over. It is a unique feature of Belle Vue that, in addition to the Nurses' Home, 426, East Twenty-sixth Street, where the women nurses reside, there is a home and training school for male nurses, who obtain their instruction in, and certificates of training from, the Belle Vue Hospital. Out of a total staff of 119 nurses, 62 are females, and 57 are males. We shall have something to say about the nurses later on, but it would be impossible to give an idea of the system of management unless we set forth these facts at the outset.

#### General Features.

The Belle Vue Hospital is a pleasant institution to visit. It is evident to the experienced eye that those responsible for its administration have succeeded in producing an *esprit* which makes its influence felt by every member of the staff from the highest to the lowest. If there be any failure in this respect, it will be found amongst the male nurses, who, though for the most part young in years, seem in a measure to lack the vivacity and earnestness of purpose, which was apparent in the case of the female nurses throughout the entire institution. Although several of the wards are not of recent construction, with one or two exceptions they are structurally well adapted to the treatment of patients who are seriously ill. It is evident that the medical staff are fully alive to the importance of securing the maximum of fresh air and sunlight in all wards, whilst they recognise the dangers of overcrowding by keeping the number of beds everywhere down to the proper limit. This encouraging fact is testified by the circumstance that none of the wards contain end beds, and that all the newest hygienic principles have evidently been noted and enforced wherever possible throughout the institution. In astounding contrast to the wisdom thus displayed by the honorary medical staff stands the ludicrous enactment of some district medical officer of health, who has divided one of the children's wards into three groups of beds by placing two pieces of tape across the ward, with the object, as explained to us, to secure that if one of the children fell ill with scarlet fever it could be isolated from the rest by being placed on the other side of this tape in the same ward. We presume that this gentleman is the nominee of a political body, for in no other way can be explained so amazing an arrangement for accomplishing the object in view under conditions full of danger to every inmate of this ward. It has begun to be the practice for wealthy citizens to erect special wards for the treatment of cases by individual members of the medical staff who may have rendered valuable service to the donors, their family, or friends. Several of these small clinics are admirably arranged, and the work undertaken seemed to be carried out in the best and most practical manner. It is a feature of this institution that, in addition to the large operation theatre, every group of wards has a small operation room, wherein all but the most serious operations can be performed to the no small advantage of the patients and the staff. Regarded as a whole Belle Vue reminds us in may respects of the Royal Infirmary, Edinburgh, because it is quite evident that each member of the honorary medical staff takes a direct personal interest in the wards allotted to his charge, over which he exercises a beneficent influence, which is fraught with good to all concerned.

#### The Condition of the Wards.

As we have stated, most of the wards are well constructed, and the furniture is, on the whole, suitable and adequate. The whole institution was beautifully clean, and the condition of the patients, and especially of the children, many of whom come from the lowest parts of the city, reflected the greatest credit upon the nursing staff. We have seldom seen brighter or more airy wards, and the attention which is paid to ventilation and comfort was exemplified by the fact that, although the temperature in New York City was 104 in the shade on the day of our visit, we found the interior of the Belle Vue Hospital to be the coolest of all places within the city. There was a surprising absence of toys and games, a fact we earnestly commend to New Yorkers' attention. The beds are of iron, to which spring mattresses have been fixed. Upon each of these, in lieu of ordinary mattresses, folded blankets, fastened by safety-pins, are placed, an arrangement which promotes the comfort of the patients, and secures the maximum of cleanliness too. In response to an inquiry as to why felt was not substituted for the blankets, we were informed that experience showed that not only was felt more expensive, but that it proved in practice far more difficult to keep hygienically clean and satisfactory.

We noticed that the condition of the wards in the main building was infinitely better than that of those in the pavilion situated in the grounds, a circumstance which we believe to be due in no small degree to the quality of the nursing work in the two buildings. It is a remarkable fact that the charge nurses are all pupil nurses, and that no graduated nurses are employed within the hospital in any capacity, so far as we were able to ascertain. In other words, the whole of the nursing is done by probationers, who give, we understand, the maximum of satisfaction to the medical staff.

### The Lunacy Pavilion.

This temporary asylum consists of one storey, and contains about 30 cells. It is excellently constructed, and is divided into two departments, one of which is devoted to men, and the other to women. Into this building are received all the cases of insanity which occur in New York City that are brought under the notice of the police and city authorities. Patients are retained here for a few days, and, if there is no hope of a speedy recovery, they are sent in due course to one of the asylums which have been established by the Board of Charities and Correction. Any committee who contemplates the erection of a similar building would do well to communicate with the Board of Charities and to procure a plan of this building. The patients are in charge of a resident medical officer, who is assisted by three female and three male attendants. We found the pavilion in excellent order, and, judging by the appearance and condition of the patients, everything seems to be done to make their lot as happy as possible.

### The Laboratories and Dispensary.

Although we visited the store-rooms and kitchens, we do not propose to give an account of what we saw there, because new buildings are in course of erection which will no doubt enable the management to remedy certain defects which are at present apparent to the initiated. The laboratories and drug stores of the Belle Vue Hospital form one of the most pleasant features of the establishment. They are under the skilled direction of a most able physician and chemist, Dr. Charles Rice, who has devoted his life to this work. In the whole of our experience we have never found any drug stores or laboratories in such excellent order, nor have we ever seen the retorts, the floors, and other appliances of a hospital dispensary so well kept or so clean as those at Belle Vue. We believe it is not too strong a statement to say that there is probably no hospital in the world where the prescriptions are more accurately dispensed, nor one where the character and quality of the drugs are better, nor where the risks attending the dispensing of hundreds of prescriptions are so systematically reduced to a minimum. Dr. Charles Rice is widely known as a most able writer and chemist. He has exercised much self-denial in remaining so many years at a post the emoluments of which cannot be great. His work is excellent in all respects, and he deserves a large measure of public recognition for his devotion to a work which, though attractive in some respects, must often prove most laborious and exacting.

### Some Criticism and Suggestions.

We hope we may be allowed, without offence, to make a few suggestions which, if adopted, would tend in no small degree to improve the hygienic condition of this great institution. The lavatory, water-closets, slop sinks, and other sanitary fittings are out of date, and it is time that the whole of them were reconstructed and renewed by the introduction of more modern and suitable appliances. The present ones have nothing to commend them, they are old, and, for the most part, unsuitable to modern hospital requirements. The baths, too, are old and bad, and they need to be replaced by porcelain baths of the best pattern. Although there were exceptions, the wards contained but few pictures, and there was a marked absence of flowers everywhere. This is probably due to the fact that, comparatively few New York citizens take an active personal interest in the Belle Vue Hospital, and we hope that the time is not far distant when an army of the more wealthy residents will realise the privilege of rendering a little personal service to Christ's poor by paying periodical visits to Belle Vue, and leaving behind them the means to brighten and cheer the patients by the introduction of flowers, ferns, pictures, and other helpful aids to recovery. Mr. Porter would be delighted to make arrangements to take any one or more of the great millionaires and wealthier citizens, their wives and daughters, through the wards of Belle Vue. Such a visit, under his able guidance, would no doubt be fruitful in result, and prove helpful not only to the authorities and patients, but to the visitors by awakening in the latter a sense of the value of the work done, and so enabling them to enjoy the privilege of supplementing that work by liberal contributions and heartfelt personal sympathy. We were very much struck by the circumstance that the clothes of the patients are allowed to remain in the wards. It is true, we believe, that when a patient is first taken into the hospital his clothes are disinfected, but it is highly desirable to make provision outside the wards, where the patients' clothes may be put away until they are wanted for use. We have ventured to make these suggestions, because we desire to render some little return to Mr. Porter and the medical and nursing staff for their courtesy and kindness on the occasion of our visit. New York has good cause to be proud of Belle Vue Hospital, and we certainly hope that many citizens will take an opportunity of visiting the institution, because we know if they do they will share the satisfaction which we experience, and feel that by making this provision for the sick poor of the city the citizens deserve the thanks of their day and generation.

